# Rescue Cervical Cerclage : Prevention of a Previable Birth

**DOI:** 10.7759/cureus.6994

**Published:** 2020-02-14

**Authors:** Divya Pandey, Neha Pruthi Tandon

**Affiliations:** 1 Obstetrics & Gynaecology, Vardhman Mahavir Medical College and Safdarjung Hospital, New Delhi, IND; 2 Obstetrics & Gynaecology, Post Graduate Institute of Medical Education and Research, Dr. Ram Manohar Lohia Hospital, New Delhi, IND

**Keywords:** emergency cervical cerclage, cervical dilatation

## Abstract

Rescue cervical cerclage can effectively prolong a nonviable gestation to viability, if done correctly in chosen patients after appropriate counseling. Here, we present a case study of an antenatal woman with advanced cervical changes at 24 weeks who benefited from the rescue cervical cerclage procedure to have a successful pregnancy outcome.

## Introduction

The benefits of rescue cervical cerclage (also known as emergency cerclage/stitch or rescue stitch) have been controversial [1]. It is a rescue procedure to prolong pregnancy to a viable gestation in women presenting in the second trimester with cervical dilatation and bulging fetal membranes. Under emergency circumstances, when done with all aseptic precautions, it can significantly prolong pregnancy and increase the chance of viable pregnancy outcomes. However, the increased risk of infection due to exposure of the fetal membranes to vaginal bacteria has to be explained to the patient. Rescue cerclage can be offered to women without signs of infection, active vaginal bleeding and active labor [2].

 This paper will discuss a case of successful cerclage done in the second trimester at 24 weeks.

## Case presentation

**Figure 1:**
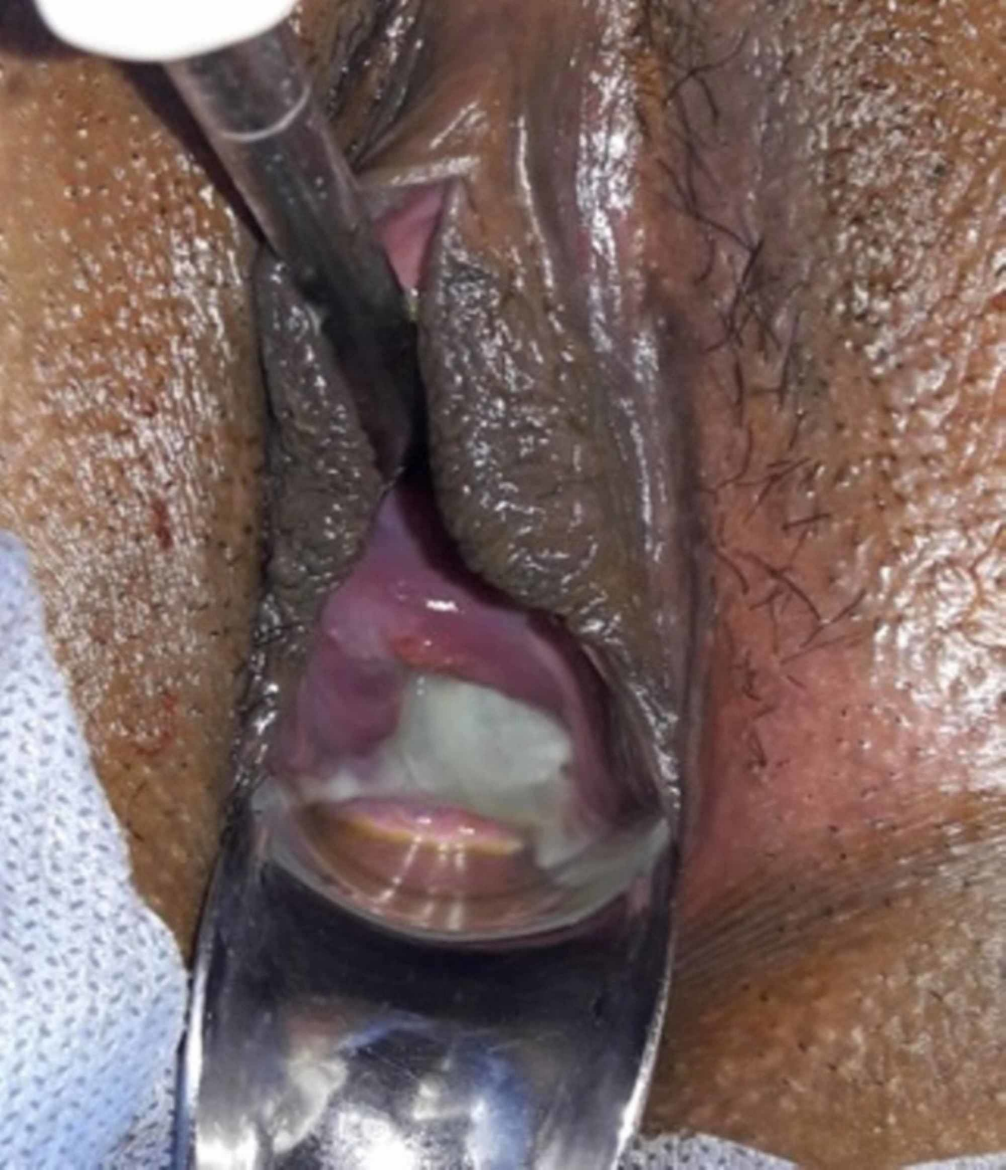
Photograph showing bulging membranes through well-effaced cervix

A 26-year-old female with a previous history of one miscarriage at 22 weeks presented to the emergency at 24 weeks of gestation with heaviness in the lower abdomen. There was no history of watery discharge /bleeding per vaginam. There was no history suggestive of urinary tract infection. Her general condition was fair and vitals were stable. Fundal height corresponded to the period of gestation. On per speculum examination cervix was 4 cm dilated with membranes bulging through the external cervical os into the vagina (figure 1). The patient was admitted, investigations [complete hemogram, urine routine and microscopic, C-reactive protein (CRP), cervical culture] were sent, steroid (dexamethasone) and tocolytics (isoxsuprine infusion) were started. Ultrasound showed a single live fetus corresponding to 24 weeks with fundal placenta and an effective fetal weight of 900 grams. The patient was explained all the risks and benefits of the emergency cerclage and written consent was taken for the procedure.

**Figure 2:**
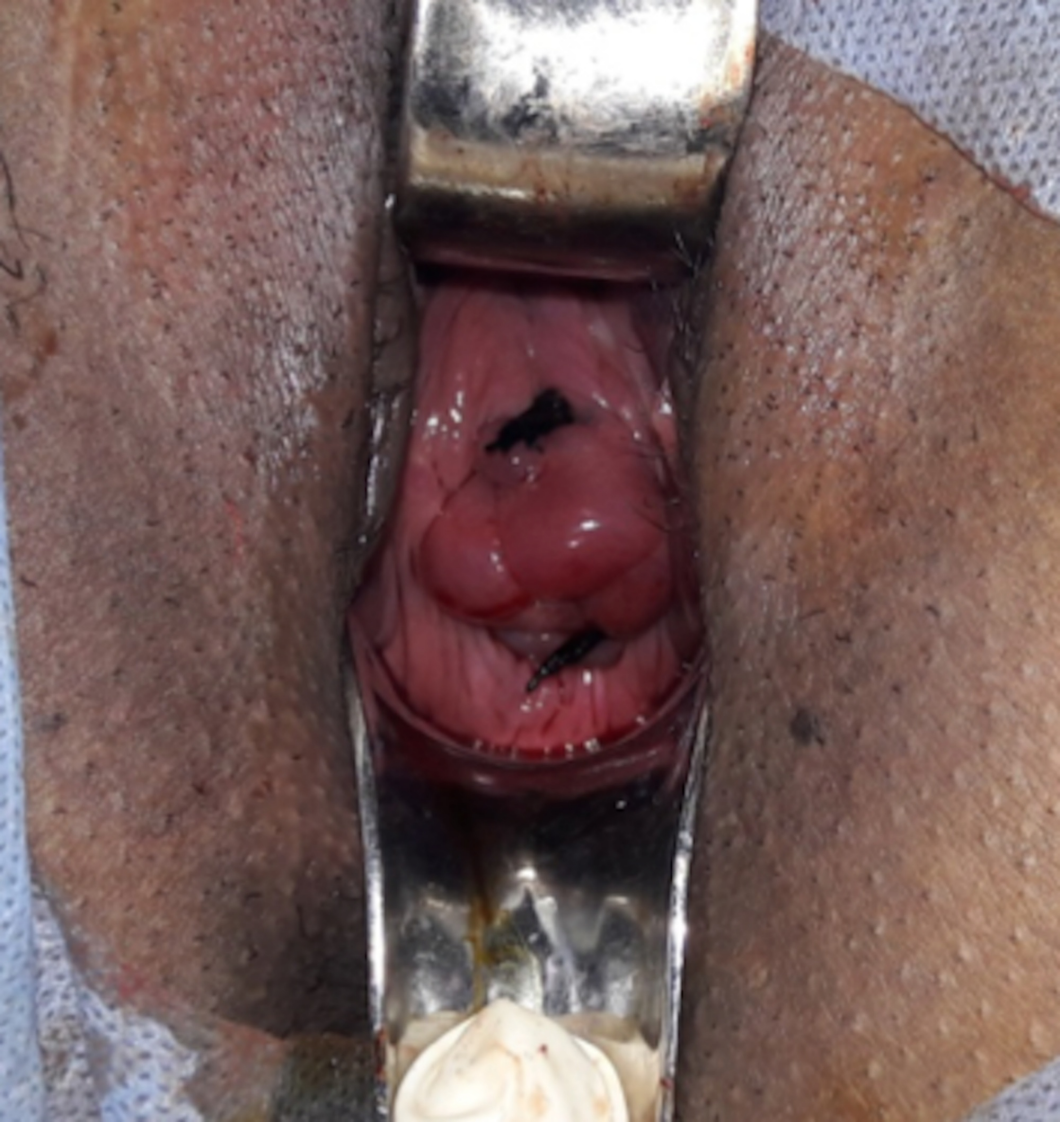
Photograph showing cerclage in situ. In view of well taken up cervix, Wurm's Stitch has been applied after repositioning bulging membranes

Emergency cerclage was planned under spinal anesthesia. A foleys bulb inflated with 15 ml saline was used to reposit the bulging membranes. Wurm's stitch was applied (as the cervix was well effaced) using silk under aseptic conditions (figure 2). Two stitches were applied at 12 o' clock and 6 o' clock position (Figure 2). In the postoperative period, the patient was administered antibiotics and tocolysis (intramuscular isoxsuprine injection, 10 mg 6 hourly for 48 hours then gradually tapered to 8 hourly injection and then switched to oral isoxsuprine tablet for one week, then stopped along with intramuscular 17-alpha hydroxyprogesterone caproate injection once a week ). The patient was followed with weekly CRP and total leucocyte counts. Report for cervical culture was negative. The patient was discharged at 26 weeks of gestation on weekly intramuscular 17-alpha hydroxyprogesterone caproate injection as per recommendation by the Society of Maternal Fetal Medicine. Routine maternofetal surveillance was followed. The pregnancy thrived well till 34+5 weeks when she developed premature rupture of membranes (PROM). Preterm fetus of 2.0 kg was delivered vaginally. The neonate stayed in Neonatal Intensive Care Unit (NICU) for two days and thereafter was discharged.

## Discussion

Women presenting with painless cervical dilatation in second trimester are left with two options of management: one is expectant and the other is rescue cervical cerclage. Although there are limited randomized controlled trials and meta-analysis on expectant bed rest vs rescue cerclage to date, a few observational studies have shown that pregnancy is prolonged by 6 to 9 weeks with rescue cerclage compared to less than 4 weeks with expectant management (bed rest) [3-6].

Namouz et al. conducted a literature review in 2013 including 34 studies, which included majority of observational and limited randomized controlled trials. Their data suggested that rescue cerclage was associated with a longer latency period and better pregnancy outcomes when compared with bed rest [7].

Stupin et al. conducted a retrospective trial on 161 women with amniotic sac prolapse. Improved perinatal outcome-live birth rate, birth weight was seen in the cerclage group [4]. Smaller observational trials and retrospective studies have found significantly increased interval from treatment‑to‑delivery, increased mean birth weight, higher neonatal survival rates, and live birth rates with decreased NICU stay in the emergency cerclage groups [3, 8]. In another study by Olatunbosun et al., it was found that women treated with cerclage required a significantly shorter period of antepartum hospitalization, decreased use of tocolytics, and experienced less preterm membrane ruptures compared to women in the bed rest group. There was no statistical difference in the frequencies of chorioamnionitis, maternal morbidity or cesarean section between the two groups [6].

In the present case, there was increased treatment to delivery interval by 10 weeks and an increase in weight by 1.1 Kg. This case adds to the existing data on women undergoing cerclage. Rescue cerclage is a favorable approach in women with cervical dilatation in the second trimester.

According to the Society of Obstetrics and Gynecology of Canada (SOGC) guidelines, emergency cerclage may be considered in women in whom the cervix has dilated to < 4 cm without contractions before 24 weeks of gestation [9]. Recent National Institute of Clinical Excellence (NICE) guidelines recommend that rescue cervical cerclage should be considered for women between 16 and 27 weeks with a dilated cervix and exposed unruptured fetal membranes. However, benefits of cervical cerclage are more when applied at earlier gestations [2].

## Conclusions

Rescue cervical cerclage is a safe and easy surgical procedure that can prolong pregnancy to viability even with advanced cervical changes. This procedure should be undertaken in an antenatal woman with advanced cervical changes after analyzing the overall clinical picture and comprehensive counseling.
